# Supplementary figures and images for: Bypass of Dfi1 Regulation of Candida albicans Invasive Filamentation by Iron Limitation
Source: mSphere. 2022 Feb 2;7(1):e00779-21. doi: 10.1128/msphere.00779-21 (PMC8809383; doi:10.1128/msphere.00779-21)

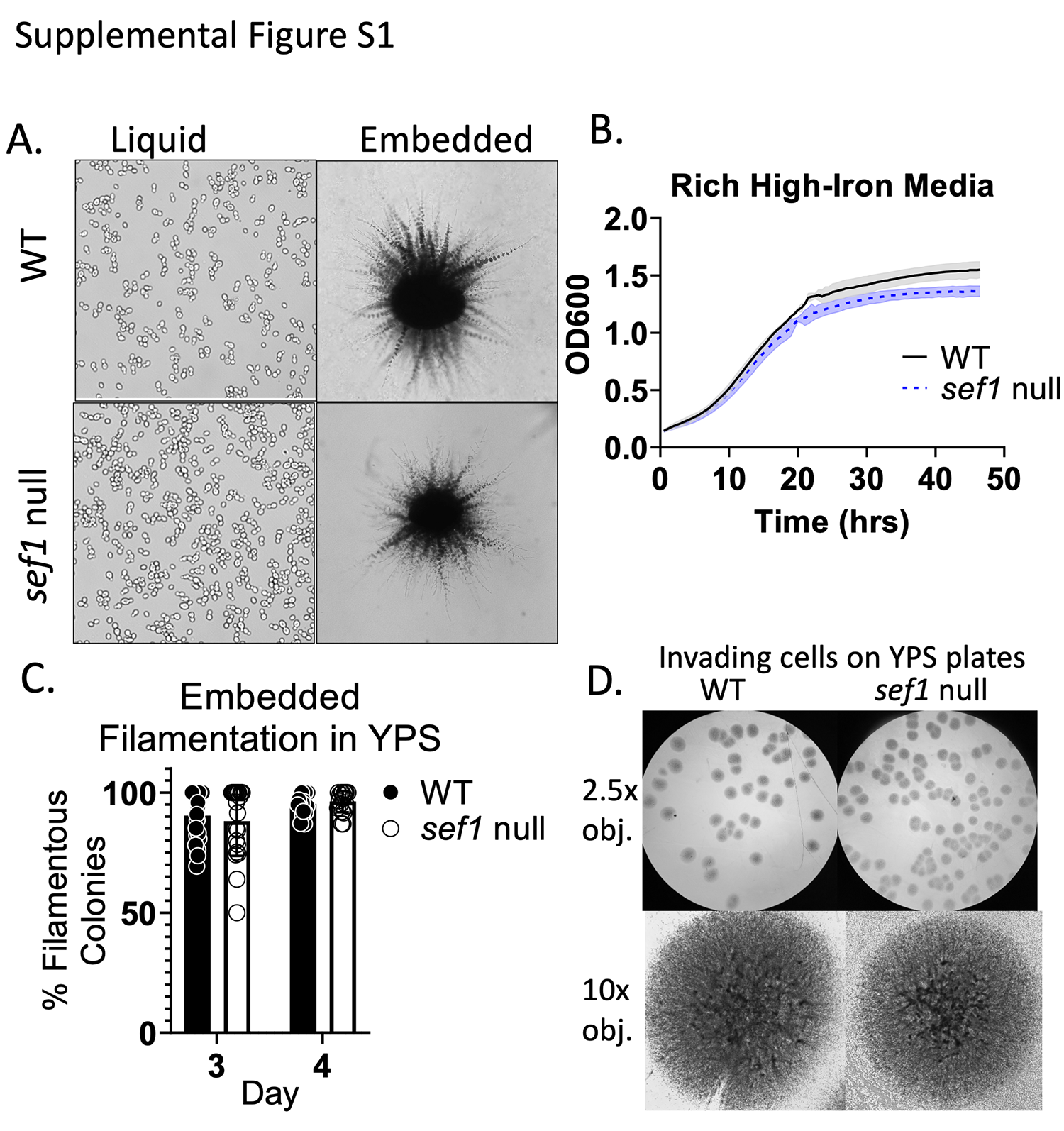

Supplement: FIG S1 [file msphere.00779-21-sf001.tif]

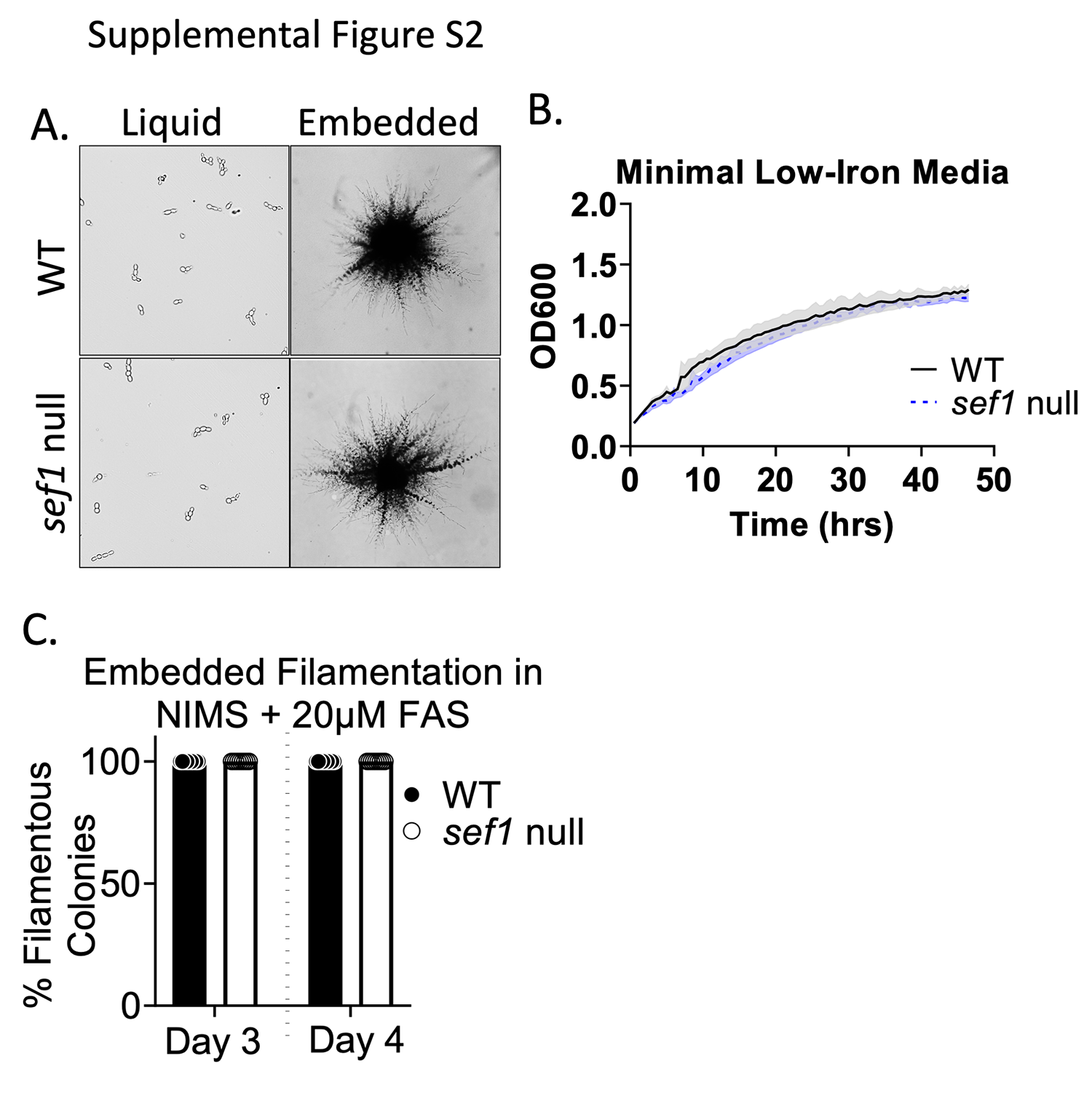

Supplement: FIG S2 [file msphere.00779-21-sf002.tif]

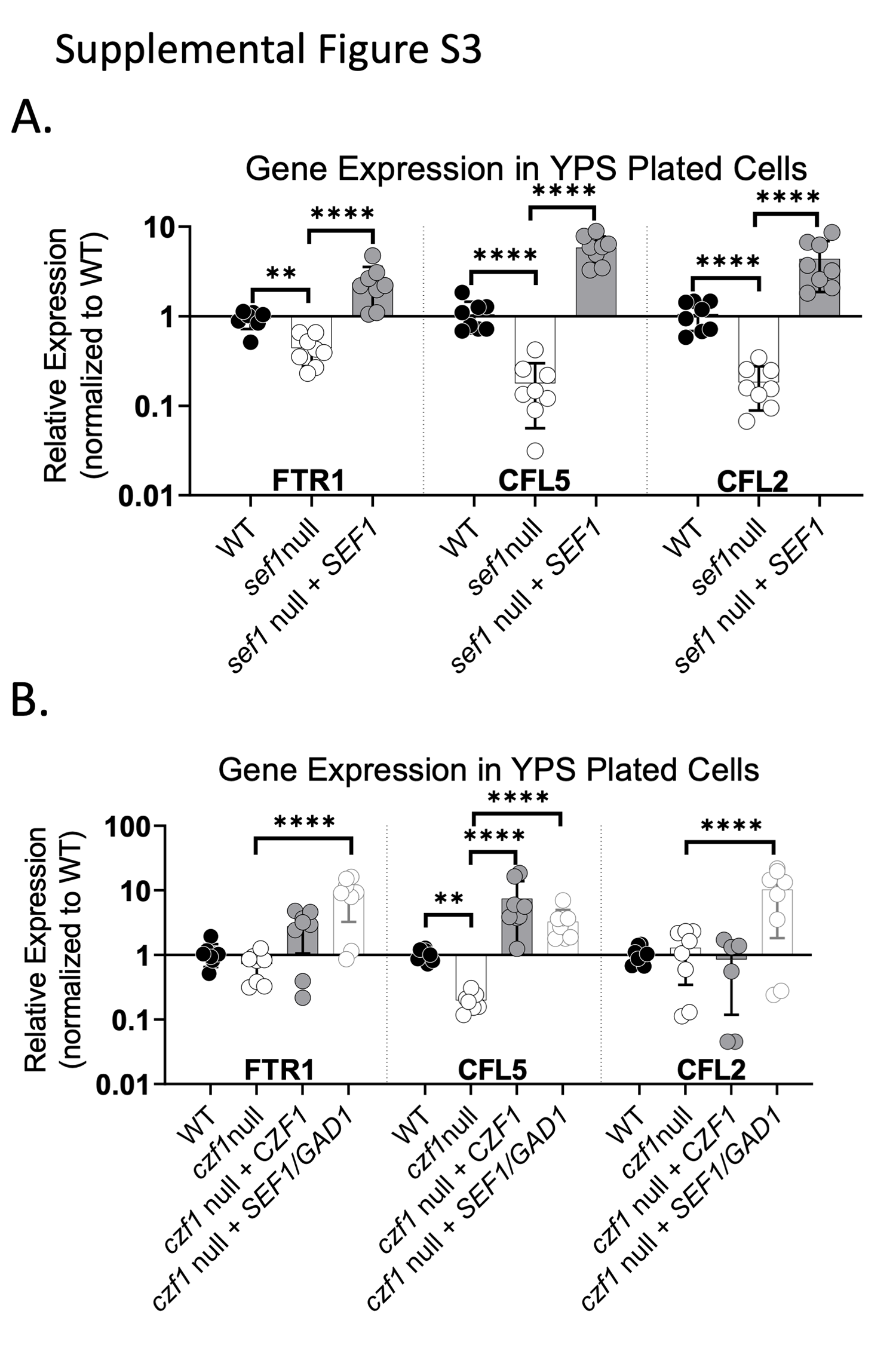

Supplement: FIG S3 [file msphere.00779-21-sf003.tif]

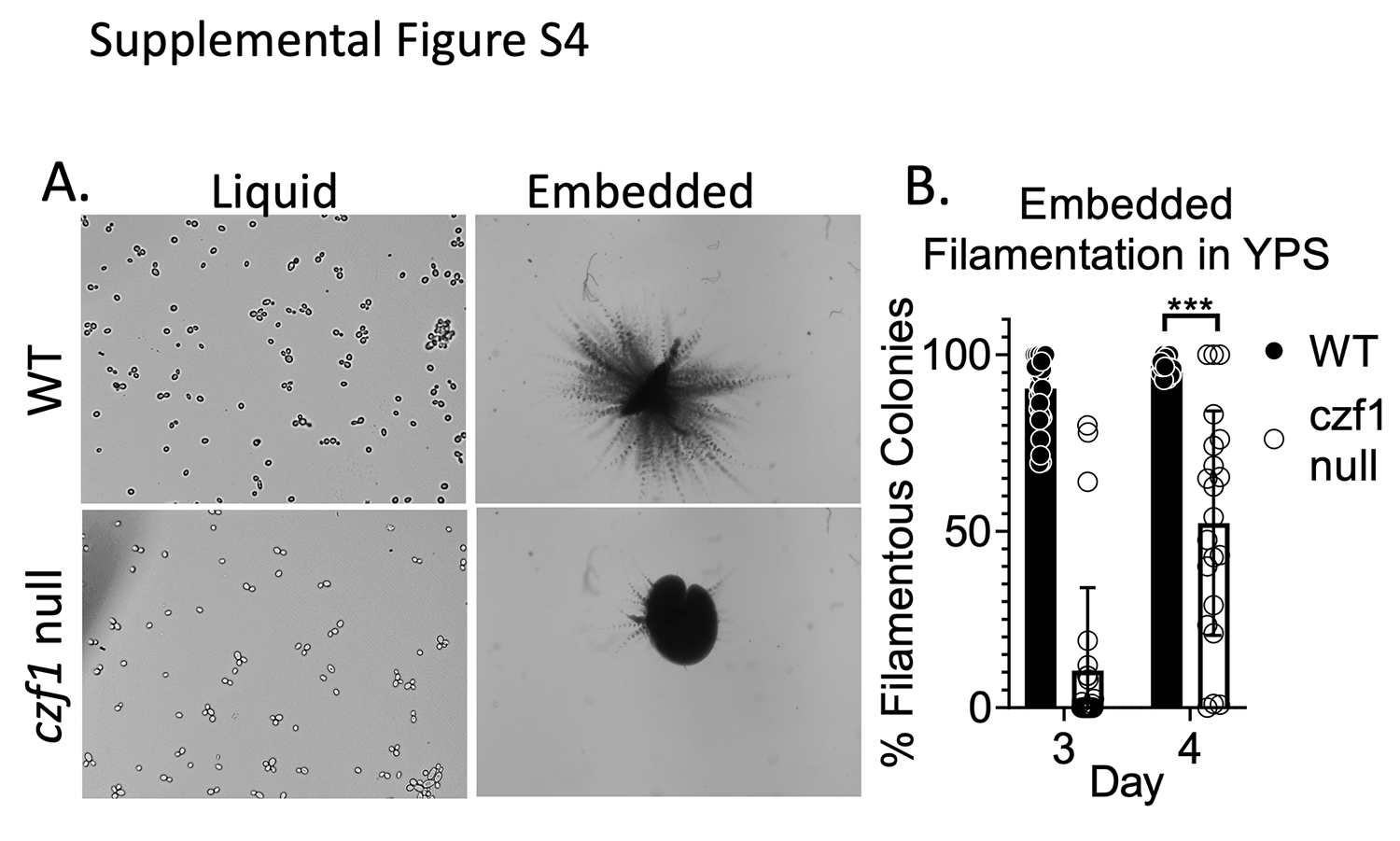

Supplement: FIG S4 [file msphere.00779-21-sf004.tif]

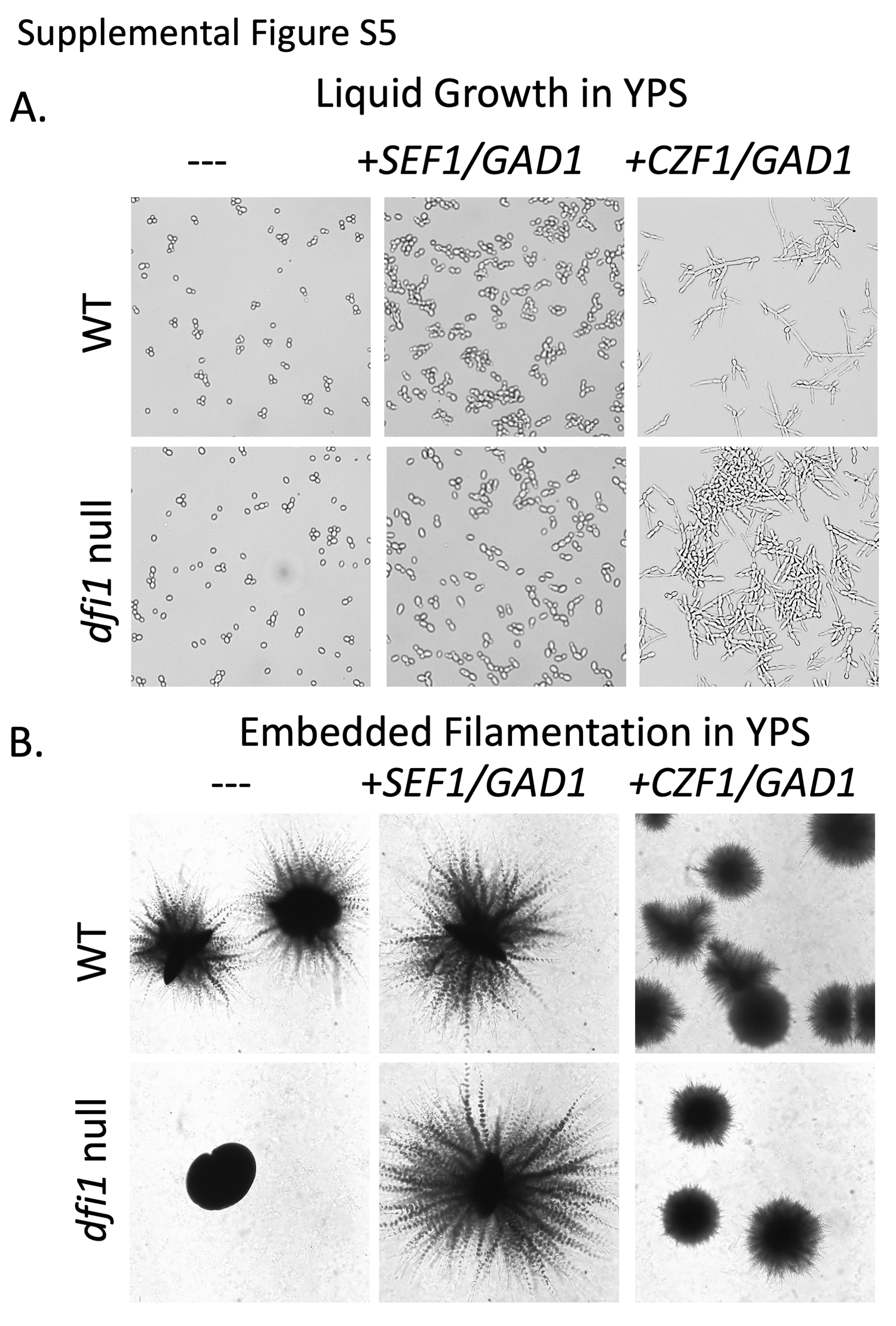

Supplement: FIG S5 [file msphere.00779-21-sf005.tif]

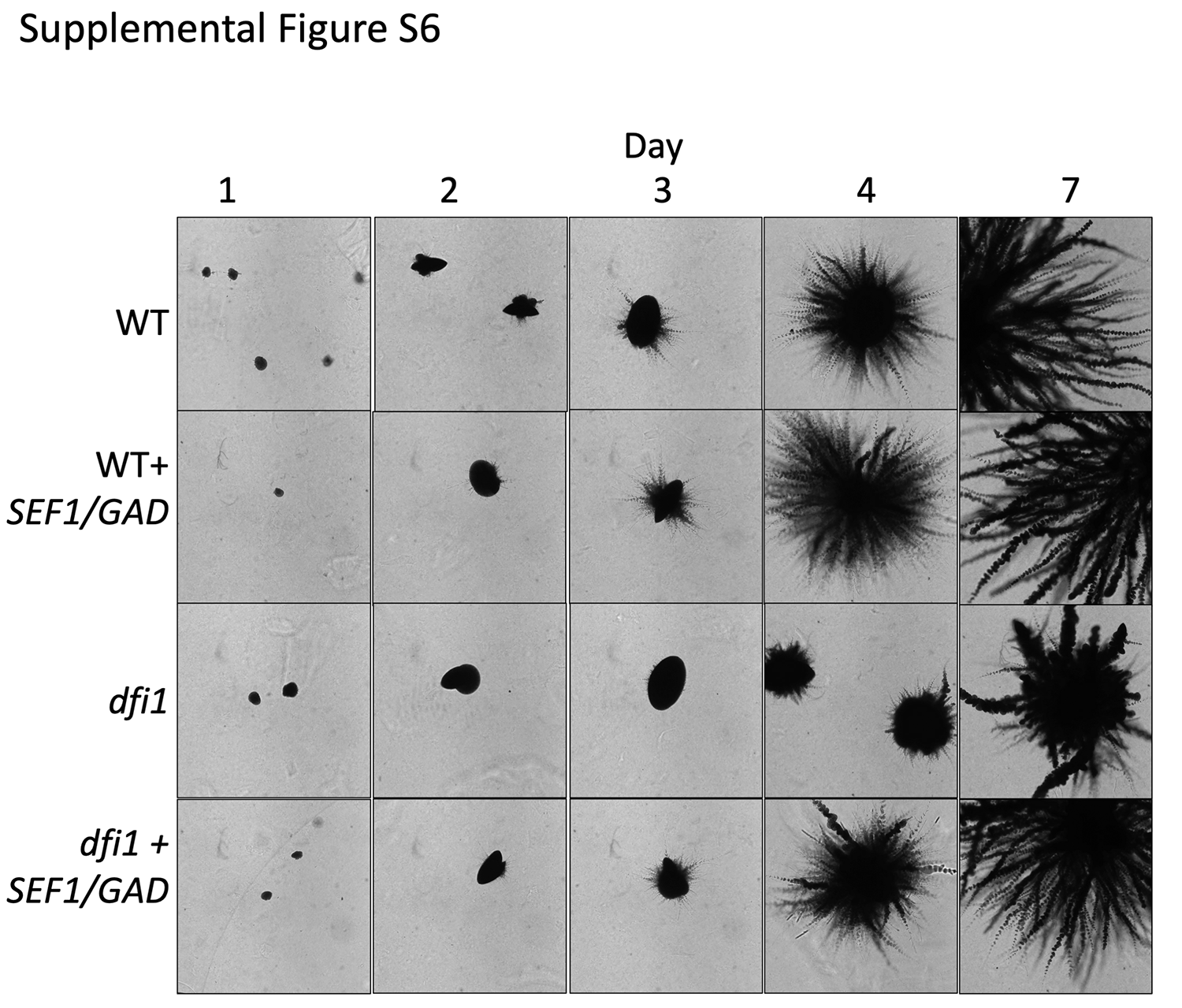

Supplement: FIG S6 [file msphere.00779-21-sf006.tif]

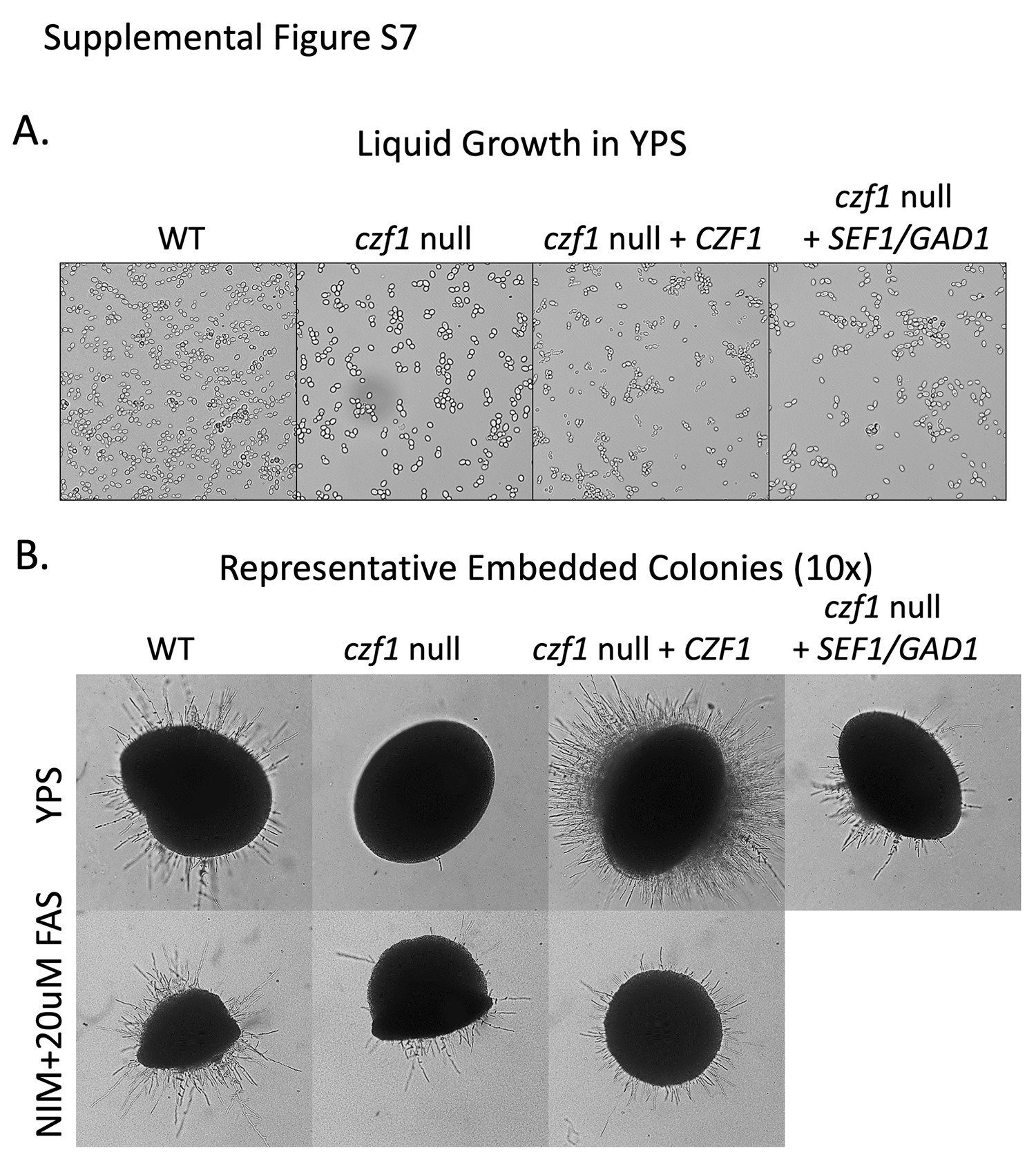

Supplement: FIG S7 [file msphere.00779-21-sf007.tif]

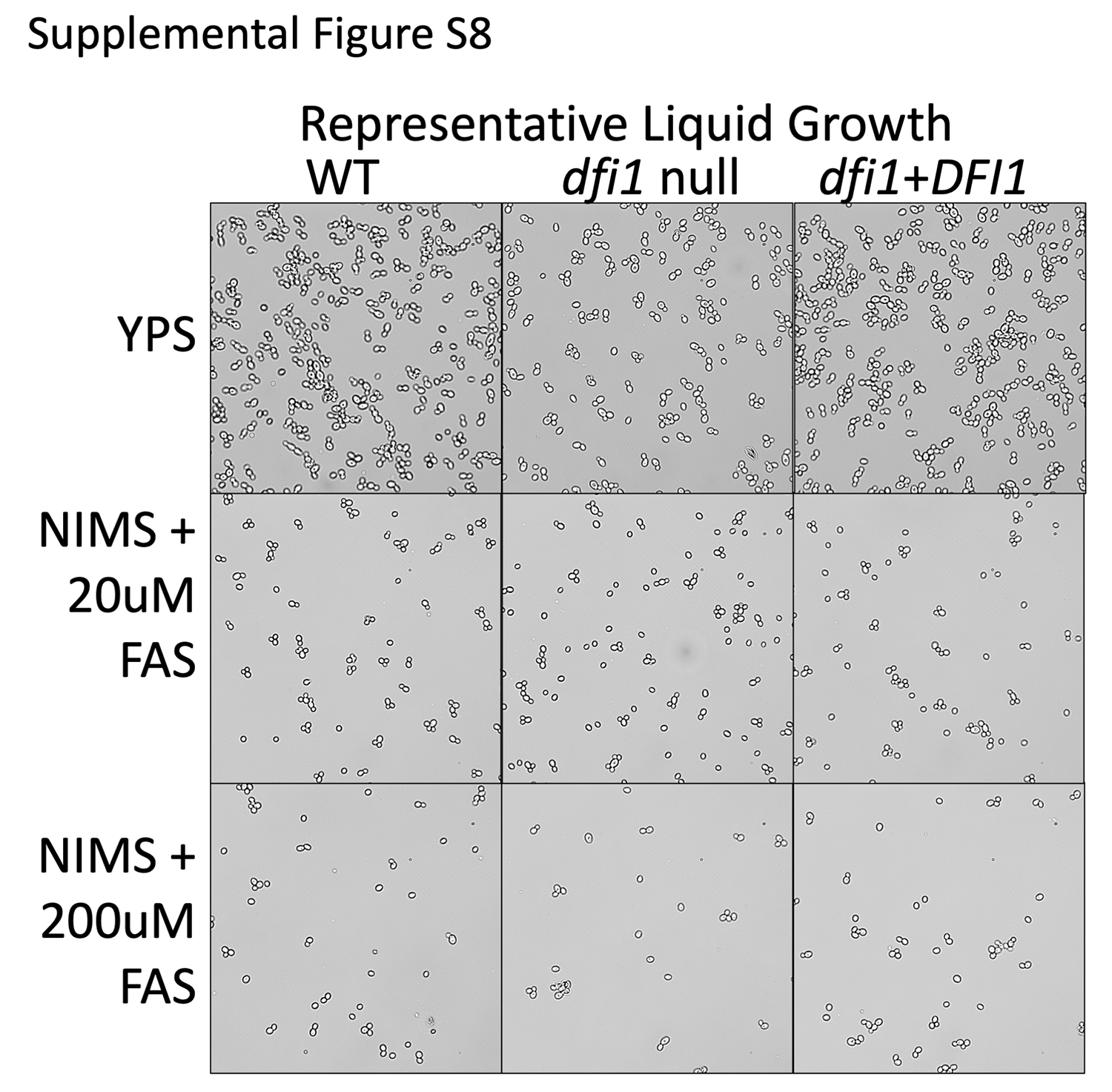

Supplement: FIG S8 [file msphere.00779-21-sf008.tif]
