# Supplementary material for: Bypass of Dfi1 Regulation of Candida albicans Invasive Filamentation by Iron Limitation
Source: mSphere. 2022 Feb 2;7(1):e00779-21. doi: 10.1128/msphere.00779-21 (PMC8809383; doi:10.1128/msphere.00779-21)
Supplement: TABLE S1 [file msphere.00779-21-st001.docx]

**Table S1: List of Strains Used in This Study**

| **Strain** | **Description** | **Genotype** | **Source** |
| --- | --- | --- | --- |
| pcz1 | wildtype | BWP17, *ura3*Δ::*imm434*/*URA3* | Zucchi et al, 2011 |
| pcz5 | *dfi1* null | Pcz1, *dfi1Δ/dfi1Δ* *ura3*Δ::*imm434/URA3* | Zucchi et al 2011 |
| pcz9 | *dfi1*/*DFI1* | pcz5, *dfi1*Δ/*dfi1*::*DFI1*-His_6_HA-HIS placer | Zucchi et al 2011 |
| SN425 | wildtype | SN152, *his1∆/HIS1, leu2∆/LEU2* | Homann et al, 2009 |
| TF015-Y | *sef1* null | SN152, *sef1*∆::*HIS1/sef1*∆::*LEU2* | Homann et al, 2009 |
| TF104-X | *czf1* null | SN152, *czf1*∆::*HIS1*/*czf1*∆::*LEU2* | Homann et al, 2009 |
| arj1 | WT+*SEF1*/GAD | pcz1, *ADH1/adh1*::P*_ADH1_*-*SEF1-GAL4AD*-HA-*caSAT1* | This work |
| arj2 | *dfi1*+*SEF1*/GAD | pcz5, *ADH1/adh1*::P*_ADH1_*-*SEF1-GAL4AD*-HA-*caSAT1* | This work |
| arj3 | WT+*CZF1*/GAD | pcz1, *ADH1/adh1*::P*_ADH1_*-*CZF1-GAL4AD*-HA-*caSAT1* | This work |
| arj4 | *dfi1*+*CZF1*/GAD | pcz5, *ADH1/adh1*::P*_ADH1_*-*CZF1-GAL4AD*-HA-*caSAT1* | This work |
| arj5 | *sef1* null + *SEF1* | SN152, *sef1*∆::*HIS1/sef1*∆::*LEU2::* P*_ADH1_*-*SEF1* -HA-*caSAT1* | This work |
| arj6 | *czf1* null + *CZF1* | SN152, czf1∆::HIS1/czf1∆::LEU2:: P*_ADH1_*-*CZF1*-HA-*caSAT1* | This work |
| arj7 | *czf1* null + *SEF1/GAD* | SN152, *czf1*∆::*HIS1*/*czf1*∆::*LEU2::* P*_ADH1_*-*SEF1-GAL4AD*-HA-*caSAT1* | This work |
