# Supplementary material for: Bypass of Dfi1 Regulation of Candida albicans Invasive Filamentation by Iron Limitation
Source: mSphere. 2022 Feb 2;7(1):e00779-21. doi: 10.1128/msphere.00779-21 (PMC8809383; doi:10.1128/msphere.00779-21)
Supplement: TABLE S2 [file msphere.00779-21-st002.docx]

**Table S2: List of Primers Used in This Study**

| **Gene** | **Primer Name** | **Sequence** | **Purpose** | **Source** |
| --- | --- | --- | --- | --- |
| *ACT1* | AJ_ACT1_F | GTTGGTGATGAAGCCCAATC | qPCR | This work |
|  | *AJ_ACT1_R* | CCCAGTTGGAAACAATACCG | qPCR | This work |
| *CFL5* | *AJ_CFL5_F* | CCAACAGTTGCTGTTTGGTG | qPCR | This work |
|  | AJ_CFL5_R | *GCCTTAGGGAATCCGAAAAC* | qPCR | This work |
| *SOD4* | *AJ_SOD4_F* | *AGCCAGTTCCAGAATCCAAA* | qPCR | This work |
|  | *AJ_SOD4_R* | *CAGCTGGAGTTTTGGCAGTA* | qPCR | This work |
| *OPT1* | AJ_OPT1_F | *CGTTAAAGAACATACTATCATCACTATT* | qPCR | This work |
|  | *AJ_OPT1_R* | *GTACTCCAGATAAGTAATAAATTGTACC* | qPCR | This work |
| *BMT9* | AJ_BMT9_F | *TCAGAAACCATGTATGCACCA* | qPCR | This work |
|  | *AJ_BMT9_R* | *CCACATTTTTCCCCTCACAT* | qPCR | This work |
| *CSA1* | *AJ_CSA1_F* | *CAGCTAACGTGCAAACGAGT* | qPCR | This work |
|  | *AJ_CSA1_R* | *GATTCGGAAGCAGAAGCAAC* | qPCR | This work |
| *GDH3* | *AJ_GDH3_F* | *GTAAAGAAACTTTCAAAGGTAAAAGAG* | qPCR | This Work |
|  | *AJ_GDH3_R* | *CATTCTTAGAAATGATTGAACCTTTAGA* | qPCR | This Work |
| *CFL1* | *AJ_CFL1_F* | *TAAATATAATACTCGTATGAAACGTGAC* | qPCR | This Work |
|  | *AJ_CFL1_R* | *ATTATGGATCAAAAAGAAGACTTCATAA* | qPCR | This Work |
| *CFL2* | *AJ_CFL2_F* | *ATTAATTTTATTTGGTGGTAGAAACAAC* | qPCR | This Work |
|  | *AJ_CFL2_R* | *GAGAAATCCATCTGTGATACATAATAAA* | qPCR | This Work |
| *FET31* | *AJ_FET31_F* | *AATTTATATTTGATCAATGGTTTCGATG* | qPCR | This Work |
|  | *AJ_FET31_R* | *TGATCATCAACAGTAAAATTGTATAAGA* | qPCR | This Work |
| *FTR1* | *AJ_FTR1_F* | *TTTATTATCTGTGTTTTGTTGTTAATG* | qPCR | This Work |
|  | *AJ_FTR1_R* | *GCTTTGTTTAATTCTAACTTCTTCTTA* | qPCR | This Work |
| *MRS4* | *AJ_MRS4_F* | *TTTGATATGTTAAAACAAAGAATGCAAG* | qPCR | This Work |
|  | *AJ_MRS4_R* | *GCTTTATAGATATCTGATGCTAATTTGA* | qPCR | This Work |
| *FET33* | *AJ_FET33_F* | *TTAAATAATATGGATCCAGGTAAACATC* | qPCR | This Work |
|  | *AJ_FET33_R* | *ATGTTTCATTTGTAGGATCAAATACTAA* | qPCR | This Work |
| *FTR2* | *AJ_FTR2_F* | *TTTTCATCATTTCTGTTTTGTTATTGAT* | qPCR | This Work |
|  | *AJ_FTR2_R* | *TGTTTAATTCTAACTTCTTCTTACCTTT* | qPCR | This Work |
| Sef1 | Sef1_PV5_Y | *AAATTTACAACGGACGATGC* | Confirming Deletion | Homann et al, 2009 |
|  | Sef1_INT3_475 | *TTGTGGGTTGGTTGGTGTAG* | Confirming Deletion | This work |
| Czf1 | czf1_PV5_X | *TTTCTGGCTCAATCCTGTCAT* | Confirming Deletion | Homann et al, 2009 |
|  | Czf1_INT3 | *GCTCGAAGAACTGGATGCTG* | Confirming Deletion | Homann et al, 2009 |
| *LEU2* | Leu2_R | *GAGAGTGGCCAAATGAACCT* | Confirming deletion | This work |
| *SEF1* | *AJ_SEF1_R* | *TGGCAAACTCACCGTTACCA* | Confirming presence of SEF1-GAD allele | This work |
| *CZF1* | *AJ_CZF1_R* | *ATGCACAGAATCCCGCAGAA* | Confirming presence of CZF1-GAD allele | This work |
| *ADH1* | *AJ_ADH1_F* | *CACCACAACACAACACCCAGTTT* | Confirming presence of GAD allele | This work |
